# Giemsa-Stained Wet Mount Based Method for Reticulocyte Quantification: A Viable Alternative in Resource Limited or Malaria Endemic Settings

**DOI:** 10.1371/journal.pone.0060303

**Published:** 2013-04-02

**Authors:** Wenn-Chyau Lee, Bruce Russell, Yee-Ling Lau, Mun-Yik Fong, Cindy Chu, Kanlaya Sriprawat, Rossarin Suwanarusk, Francois Nosten, Laurent Renia

**Affiliations:** 1 Department of Parasitology, Faculty of Medicine, University of Malaya, Kuala Lumpur, Malaysia; 2 Department of Microbiology, Yong Loo Lin School of Medicine, National University of Singapore (NUS), National University Health System (NUHS), Singapore, Singapore; 3 Shoklo Malaria Research Unit (SMRU), Mae Sot, Tak Province, Thailand; 4 Singapore Immunology Network (SIgN), A*STAR, Biopolis, Singapore, Singapore; 5 Mahidol-Oxford-University Research Unit, Bangkok, Thailand; 6 Centre for Tropical Medicine, University of Oxford, Churchill Hospital, Oxford, United Kingdom; State University of Campinas, Brazil

## Abstract

The quantity of circulating reticulocytes is an important indicator of erythropoietic activity in response to a wide range of haematological pathologies. While most modern laboratories use flow cytometry to quantify reticulocytes, most field laboratories still rely on ‘subvital’ staining. The specialist ‘subvital’ stains, New Methylene Blue (NMB) and Brilliant Crésyl Blue are often difficult to procure, toxic, and show inconsistencies between batches. Here we demonstrate the utility of Giemsa's stain (commonly used microbiology and parasitology) in a ‘subvital’ manner to provide an accurate method to visualize and count reticulocytes in blood samples from normal and malaria-infected individuals.

## Introduction

Patient reticulocyte profiles provide important data on erythropoietic activity, reticulocyte release into peripheral circulation and erythrocyte maturation rate. Rapid and objective reticulocyte counts by way of flow cytometry (using florescent stains such as Thiazole Orange) [1]–[2] has largely replaced microscopic examination using ‘subvital’ –––reticulocyte stains such as New Methylene Blue (NMB) and Brilliant Crésyl Blue [2]–[4]. However, the use of flow cytometry in developing countries and field laboratories is problematic due to the high cost of the equipment, limited access to servicing and unreliable power supply [5]. Perhaps the most significant confounder facing flow cytometry performance in the developing world is the possibility that a patient sample contains intraerythrocytic malaria parasites [6]. Therefore reticulocyte counts conducted in laboratories of malaria endemic areas are still reliant on the traditional microscopic method of subvital staining with a commercial NMB solution. Unfortunately, NMB is a specialist stain with limited shelf life and inconsistent resupply. Moreover, NMB stains produced by different manufacturers as well as inter-batch variations can yield significant inconsistency in reticulocyte identification [7]. Therefore, there is a need to develop an alternative staining methodology for microscopic reticulocyte quantification that is inexpensive and accurate. One of the most commonly encountered stains in the field is Giemsa's stain (Giemsa). This inexpensive purple stain is used for a range of histological and microbiological applications (i.e. identification of *Chlamydia* spp., Spirochetes and Trypanosomes) as well as its important use in malaria diagnosis. Here we compare a wet mounting method for subvitally stained Giemsa and NMB for the detection and quantification of reticulocytes in a malaria endemic field setting.

## Materials and Methods

### Blood sample collection, processing and ethics

Blood samples from infected and uninfected donors were collected after obtaining written informed consent following ethical guidelines in the approved protocols; OXTREC 027-025 (University of Oxford, Centre for Clinical Vaccinology and Tropical Medicine, UK) and MUTM 2008-215 from Ethic committee of Faculty of Tropical Medicine, Mahidol University; with specific provision for the samples used in this study. Blood samples were collected into the BD Vacutainer® with Lithium Heparin Anticoagulant. All samples were processed freshly upon collection or stored at 4°C for not more than 24 hours prior to processing.

In earlier studies we determined that reticulocyte counts were not affected by wet mounting of NMB subvital stained blood samples relative to the standard smearing of the same sample, thus allowing the direct comparison of the NMB-wet mount method with the Giemsa-wet mount method, as detailed below.

### NMB wet mount method

A 50 µl blood samples were subvitally stained with 50 µl NMB (Cat#R4132; Sigma-Aldrich®)After incubation over a period of 10 minutes at room temperature, the stained blood cells were centrifuged and the pellet was resuspended at 5% hematocrit with plain McCoy's 5A medium (RPMI medium can be substituted). Subsequently 7.5 µl of the stained suspension was dropped onto a glass slide and then covered by a 22×32 mm (0.17 mm thickness) glass cover slip.

### Giemsa wet mount method

Another 50 µl of each unstained blood sample was stained subvitally with 2.5 µl of filtered Giemsa (Cat#G4507; Sigma-Aldrich®.) for 15 minutes at room temperature, followed by gentle mixing. The wet mount was prepared as described above for the NMB.

### Reticulocyte counting

All blood smears and wet mounts were examined immediately with light microscope under oil immersion magnification. Reticulocyte percentage number was determined by counting the number of reticulocytes per 1000 erythrocytes [8].

### Statistical Analysis

Comparison of reticulocyte counts (percentage of reticulocytes in 1000 erythrocytes counted) between the NMB-wet mount method and Giemsa-wet mount method was performed using Pearson correlation and Bland-Altman analysis [9]. The relationship between the mean percentage reticulocyte count and parasite species (*P. vivax* and *P. falciparum*) were compared with an unpaired Student's t-test. Welch's correction was not necessary for this t-test as the F- test showed that the variances were not significantly different (F = 1.4, p = 0.27)

## Results

A total of 103 malaria patient blood samples were tested. Of these, 69 samples were from *Plasmodium vivax* infections, 32 cases of *Plasmodium falciparum*, 1 mixed infection of *P. vivax* and *P. falciparum*, and 1 case of *Plasmodium malariae* infection. The highest reticulocyte count based on the average of the two methods was 6.45%, the lowest being 0.07%.

**Figure 1:**
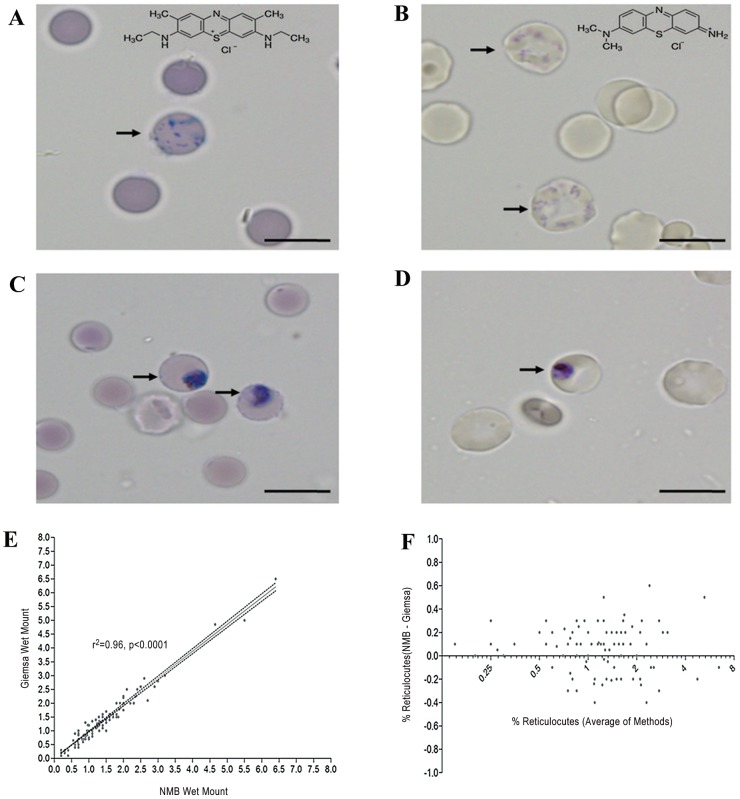
Wet mounted erythrocytes subvitally stained with New Methylene Blue (NMB) (A). A reticulocyte containing the dark reticular matter is indicated by an arrow. The chemical structure of NMB is inserted at the top right hand corner. Wet mounted erythrocytes subvitally stained with Giemsa (B). Two reticulocytes are indicated by arrows. The chemical structure of Giemsa is inserted at the top right hand corner. Wet mounts of *Plasmodium vivax* (trophozoite stage) infected erythrocytes subvitally stained with NMB (C) and Giemsa (D). The parasitized red cells are indicated by the arrows. The horizontal scale bar at the bottom right of each photomicrograph represents 10 µm. Linear Regression (E) and Bland-Altman (F) comparison of the percentage of reticulocytes (number of reticulocytes per 1000 erythrocytes) detected by Geimsa and NMB wet mount methodologies.

The stained precipitated reticular matter of the reticulocytes was clearly evident using either NMB (Fig 1A) or Giemsa (Fig 1B) wet mounting methods, however the latter method produced a lighter staining pattern. Importantly both methods enabled the clear differentiation between reticular and parasitic matter (Fig 1C and D). It is notable that the NMB produced a non-specific colouration (background stain) in the all the erythrocytes irrespective of their infection status or age (Fig 1A and C). The mean reticulocyte count in the *P. vivax* samples (1.3%+/−SD 1.05) was not significantly different to the *P. falciparum* isolates (1.57%+/−SD 0.88)(p = 0.19).

Both the NMB and Giemsa methods were significantly correlated (Pearson R = 0.97, 95% CI 0.8799 to 0.9934, r^2^ = 0.96, p<0.0001) (Fig 1E). Bland-Altman analysis indicated a good agreement between the two methods (95% limit of agreement −0.30% and 0.54%) with no significant pattern of bias (Bias  = 0.05 SD of Bias 0.2) (Fig 1F),

## Discussion

Approximately half the world's population resides in malaria endemic regions [10], and most of these areas suffer from severe limitations to health infrastructure. Therefore, clinical haematology in these regions is largely dependent on differential stains and microscopy rather than flow cytometry methods used in developed countries. The most widely practiced method for reticulocyte counts involves smearing subvitally stained blood on slides prior to microscopic examination, rather than using the wet mounting methods developed in this study. Unfortunately, reticulocyte assessment of NMB stained smears is complicated by the presence of artifactual ribosomal matter resulting from current or past (due to splenic pitting) malaria parasite infection. Furthermore, erythrocytes containing the early ‘ring’ forms of *Plasmodium* spp. and the degraded dead malaria parasites can be easily mistaken for reticulocytes on NMB smears. These issues are not entirely solved with the use of flow cytometry methods. Although DNA-specific stains enable exclusion of parasitized erythrocytes from the analysis [11], reticulocytes infected with malaria parasites (especially *P. vivax* merozoites post invasion) are also ‘gated out’ from the total reticulocyte population. Consequently, reticulocyte quantification from both methods may not reflect the actual haematological condition of malaria patients. So, although we had access to a field based flow cytometer (BD Accuri™ C6) we decided against a thiazole orange comparison with our Giemsa method; as we were concerned that malaria parasites would significantly confound the results.

Our use of the wet mounting method (NMB and Giemsa), kept viable malaria parasites alive during the examination period. The active movement of living parasites were easily differentiated from the static reticulate matter of reticulocytes. Dead parasites were differentiated from reticulocytes based on the darker staining pattern of the latter. In most cases, parasitic and host reticular matter possess a discernible difference in thickness, opacity, and refractive indices; light that has travelled through these entities is subjected to different degrees of optical interference. Such optical effects are further magnified by the liquid medium of wet mount, which has refractive index higher than that of air. However, such optical phenomena are not available on the dry smear. Hence, the optical differentiation of living parasites, dead parasites, and reticulocytes is best contrasted in wet mounting method. Certainly, our preference for wet mount staining concurs with earlier work that used wet mounts of subvitally stained blood from vivax malaria patients to confirm the preference of *Plasmodium vivax* for reticulocyte invasion [12].

While our results show that wet mount preparation from both staining methods (NMB and Giemsa) provided almost identical reticulocyte counts, the Giemsa method is marginally preferable to NMB due to its low cost and longer shelf life. However, Giemsa requires longer staining time (15 minutes) than NMB. Based on this study, a 5% Giemsa solution is recommended for the staining procedure.

Reticulocyte quantification with the Giemsa wet mount method has some limitations. A bright halo effect called spherical aberration may arise using this method. This optical effect is especially pronounced during the usage of cover slips with thickness that is incompatible to the objectives of light microscope [13]–[14]. Usage of immersion oil with incompatible refractive index can also induce this optical effect [13]–[14]. Nevertheless, this problem can be alleviated by using an appropriate glass cover slip (0.17 mm thickness). Nikon immersion oil type A with refractive index of 1.515 at 23°C allowed an optimal visualization with minimal spherical aberration. Another potential problem of the Giemsa-wet mount method is the cell movement that can impede cell counting. However, this problem can be solved by using the appropriate volume of suspension with glass cover slips of appropriate size in preparing wet mounts. Here, we show that 7.5 µl of cell suspension of 5% hematocrit value with cover slip of 22×32 mm (0.17 mm thickness) allows an even cell distribution with minimal cell movement. In addition, the wet mounts should not be examined on a slanted bench top. The wet mounts should be checked immediately after preparation, and counting must be performed as soon as possible. As the heat of the microscope light source accelerates water loss, cell crenation will be noted if the wet mount is examined for longer than 20 minutes. Riming the outside of the coverslip with Vaseline and storage in a cool dark place will allow for examination at a later time (<6 hours after preparation).

## Conclusion

Subvitally, Giemsa is a convenient and cost effective alternative to NMB for the characterisation of reticulocytes in resource limited or malaria endemic settings.
